# Isolation and Characterization of Three Sodium-Phosphate Cotransporter Genes and Their Transcriptional Regulation in the Grass Carp *Ctenopharyngodon idella*

**DOI:** 10.3390/ijms21218228

**Published:** 2020-11-03

**Authors:** Mei-Qin Zhuo, Wu-Hong Lv, Yi-Huan Xu, Zhi Luo

**Affiliations:** 1 Laboratory of Molecular Nutrition for Aquatic Economic Animals, Fishery College, Huazhong Agricultural University, Wuhan 430070, China; zmq@mail.hzau.edu.cn (M.-Q.Z.); lvwuhong@webmail.hzau.edu.cn (W.-H.L.); xuyihuan@webmail.hzau.edu.cn (Y.-H.X.); 2 School of Animal Science and Nutritional Engineering, Wuhan Polytechnic University, Wuhan 430023, China

**Keywords:** phosphorus transporter, transcriptional regulation, phosphorus homeostasis, vertebrates

## Abstract

It is important to explore the regulatory mechanism of phosphorus homeostasis in fish, which help avoid the risk of P toxicity and prevent P pollution in aquatic environment. The present study obtained the full-length cDNA sequences and the promoters of three SLC20 members (*slc20a1a*, *slc20a1b* and *slc20a2*) from grass carp *Ctenopharyngodon idella*, and explored their responses to inorganic phosphorus (Pi). Grass carp SLC20s proteins possessed conservative domains and amino acid sites relevant with phosphorus transport. The mRNAs of three *slc20s* appeared in the nine tissues, but their expression levels were tissue-dependent. The binding sites of three transcription factors (SREBP1, NRF2 and VDR) were predicted on the *slc20s* promoters. The mutation and EMSA analysis indicated that: (1) SREBP1 binding site (−783/−771 bp) negatively but VDR (−260/−253 bp) binding site positively regulated the activities of *slc20a1a* promoter; (2) SREBP1 (−1187/−1178 bp), NRF2 (−572/−561 bp) and VDR(615/−609 bp) binding sites positively regulated the activities of *slc20a1b* promoter; (3) SREBP1 (−987/−977 bp), NRF2 (−1469/−1459 bp) and VDR (−1124/−1117 bp) binding sites positively regulated the activities of the *slc20a2* promoter. Moreover, Pi incubation significantly reduced the activities of three *slc20s* promoters, and Pi-induced transcriptional inactivation of *slc20s* promoters abolished after the mutation of the VDR element but not SREBP1 and NRF2 elements. Pi incubation down-regulated the mRNA levels of three *slc20s*. For the first time, our study elucidated the transcriptional regulatory mechanisms of SLC20s and their responses to Pi, which offered new insights into the Pi homeostatic regulation and provided the basis for reducing phosphorus discharge into the waters.

## 1. Introduction

Aquaculture was considered to be one of the most important food-producing industries and provided enormous excellent protein sources for people over the world. However, the ever-increasing aquaculture is producing some adverse influences on the aquatic environment. One of the main concerns is the discharge of wastes, especially phosphorus. Inorganic phosphate (Pi) is essential for skeletal mineralization, oxidative response and cellular metabolism. Aquafeeds need dietary Pi addition in the amount that meets the requirement of fish. However, phosphorus is also the rate-limiting nutrient for the eutrophication of waters, and excess phosphorus will be excreted by the fish to the aquatic environment and result in eutrophication [1]. Thus, it is very important to investigate the mechanism of Pi absorption and transport in fish, which helps avoid the risk of phosphorus toxicity and prevent phosphorus loading in the aquatic environment.

Pi absorption and transport are mediated by sodium-phosphate cotransporters, containing three families of SLC17/type I NaPi, SLC34/type II NaPi and SLC20/type III NaPi. At present, the structure and functional role of the SLC34 members were established for the vertebrates; in contrast, relatively few studies have been performed for SLC20 proteins [2]. In mammals, the SLC20 members comprise SLC20A1 and SLC20A2, whose cDNA sequences and putative protein sequences have successfully been obtained [3,4]. They are expressed in various tissues and presumably function in Pi uptake and transport across the plasma membrane [2,5]. Moreover, low extracellular Pi content increased *slc20a1* and *slc20a2* expression in many cells [6,7], suggesting that they mediated the Pi homeostatic regulation [6]. However, despite their important roles in cellular Pi uptake, nothing is known about their precise regulatory mechanism at the transcription level. Elucidation of these mechanisms may help develop new pathways to increase dietary phosphorus utilization of fish and reduce phosphorus excretion into the environment.

The regions of gene promoters contain many important transcriptional regulatory elements which modulate their expression [8]. At present, the promoters of *slc20s* have been cloned in humans and mice [9,10] and some key transcriptional factors have been reported to regulate their transcriptional expression [9,11]. For example, vitamin D receptor (VDR), one member of the nuclear receptor (NR) superfamily, is important to regulate Pi homeostasis [12]. Nuclear factor-erythroid 2 p45-related factor 2 (NRF2), a central transcription factor regulating oxidative stress, functions in the uptake of extracellular Pi [13]. Sterol regulatory element-binding protein 1 (SREBP1) is an important transcriptional factor which regulates lipid metabolism [14] and vascular calcification [15,16]. However, direct evidence of VDR, NRF2 and SREBP1 mediating Pi homeostasis was absent. In the present study, we hypothesize that these transcriptional factors mediated the control of Pi homeostasis probably by directly targeting the *slc20s* promoters.

Teleost fish have approximately 30,000 species and they possess the most diverse group among the vertebrates [17,18], which is attributable to a teleost-specific whole genome duplication (TSGD) event during their evolution [19]. These duplicated genes offer a unique framework to explore the differentiation and diversification of their functions. Thus, teleost fish are very attractive models to study the gene evolution, structures and functions [20,21]. This study isolated the full-length cDNA and promoter sequences of three *slc20s* genes from grass carp (*Ctenopharyngodon idella*). Their mRNA tissue expression, transcriptional regulation and their function in Pi homeostasis were determined. Grass carp, a herbivorous freshwater fish, is one of the most important aquaculture species in the world and is selected as the experimental animals. The present study will increase our knowledge into the Pi homeostatic regulation and provide new knowledge for improving phosphorus utilization and reducing phosphorus pollution in the waters.

## 2. Result

### 2.1. Sequence Characterization of Three Slc20s

Here, we successfully obtained the full-length cDNA sequences of *slc20a1a* (Genbank No. MT561019)*, slc20a1b* (Genbank No. MT561020) and *slc20a2* (Genbank No. MT561021) from grass carp (Table 1), whose length was 2264, 2453 and 2125bp, encoding 644, 664 and 662 amino acids, respectively. We also obtained 1813, 1804 and 1775 bp sequences of *slc20a1a*, *slc20a1b* and *slc20a2* promoters from grass carp, respectively. The first nucleotides of their 5′ cDNA sequences were designated as +1. Several TFBSs, such as SREBP1, NRF2, VDR, ATF4, CEBPa, STAT3, CAAT-box and TATA-box, were predicted on *slc20s* promoter (Figure S1).

In grass carp, the amino acid (AA) sequences of SLC20A1A and SLC20A1B showed 61.9% and 60.0% identities with SLC20A2, respectively (Table 2). Moreover, the SLC20A1A, SLC20A1B and SLC20A2 sequences of grass carp shared the identities of 66.9–94.7%, 69.8–91.7% and 76.6–95.6% to those of other species, respectively (Table 2). The protein sequences of grass carp SLC20A1A, SLC20A1B and SLC20A2 possessed 11, 11 and 10 transmembrane domains (TMDs), respectively (Figure S2). Some conserved AA residues, such as D28, E55, D506, E575 and N81, were also found in grass carp SLC20s (Figure S2), and they were important for SLC20s function.

### 2.2. Slc20s mRNA Expression among the Tissues of Grass Carp

**Figure 1:**
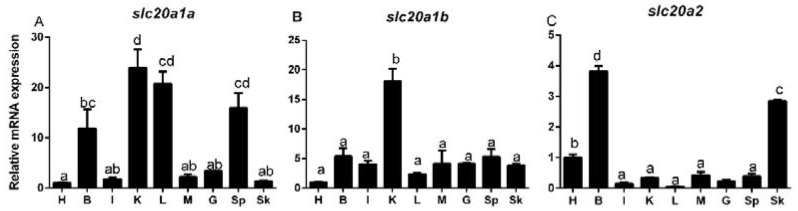
Quantitative PCR (Q-PCR) analysis for expression of *slc20a1a* (**A**), *slc20a1b* (**B**) and *slc20a2* (**C**), across heart (H), brain (B), intestine (I), kidney (K), liver (L), muscle (M), gill (G), spleen (Sp) and skin (Sk) of grass carp. Data (mean ± SEM, *n* = 3 replicate tanks. For each tank, three fish were sampled for analysis) were expressed relative to expression of housekeeping gene (*gapdh* and *18srRNA*). Expression of *slc20s* in heart was regarded as the relative expression 1. Data were subjected to one-way ANOVA and Tukey’s multiple range test. Bars that do not share a common letter mean significant difference among different tissues (*p* < 0.05).

The *slc20a1a* mRNA amounts were the highest in the kidney and liver, followed by the spleen and brain, and the lowest in other tissues. The *slc20a1b* mRNA expression was predominant in the kidney, and no significant differences were found among other tissues. The *slc20a2* mRNA amounts were predominant in the brain, followed by the skin and heart, and were lowest in other tissues (Figure 1).

### 2.3. 5′-Deletion Sequence Analysis of the Slc20s Promoters

**Figure 2:**
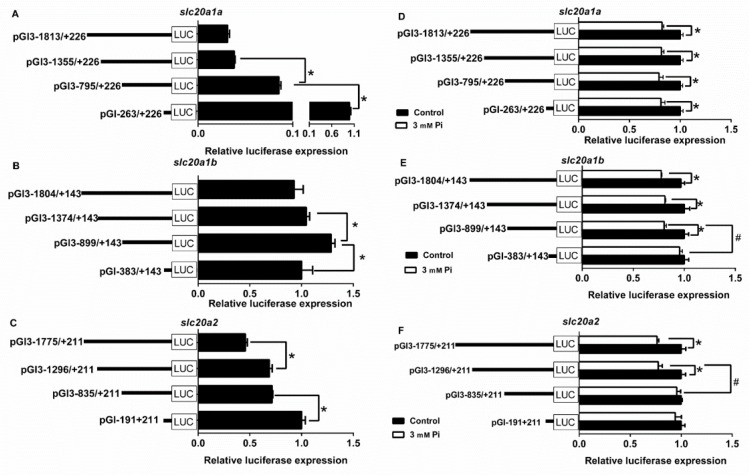
5′unidirectional deletion analysis of the *slc20a1a*, *slc20a1b* and *slc20a2* promoter regions for *Ctenopharyngodon Idella*. (**A**) A series of plasmids containing 5′ unidirectional deletions of the *slc20a1a* promoter region (pGl-1813/+226, pGl-1355/+226, pGl-795/+226, and pGl-263/+226) fused in frame to the luciferase gene were transfected into HEK293T cells. (**B**) A series of plasmids containing 5′unidirectional deletions of the *slc20a1b* promoter region (pGl-1804/+143, pGl-1374/+143, pGl-899/+143, and pGl-383/+143) fused in frame to the luciferase gene were transfected into HEK293T cells; (**C**) a series of plasmids containing 5′ unidirectional deletions of the *slc20a1a* promoter region (pGl-1775/+211, pGl-1296/+211, pGl-835/+211, and pGl-191/+211) fused in frame to the luciferase gene were transfected into HEK293T cells; (**D**) effect of Pi incubation on the promoter activity of *slc20a1a* plasmids with different length; (**E**) effect of Pi incubation on the promoter activities of *slc20a1b* plasmids with different length; (**F**) effect of Pi incubation on the promoter activities of *slc20a1b* plasmids with different length; values represent the ratio between firefly and Renilla luciferase activities. Results are expressed as the mean ± SEM arbitrary units of three independent experiments). Student’s t-test was undertaken to detect the differences between two treatments. Symbol (*) indicates significant differences between the two group (*p* < 0.05). Hash symbol (#) indicates significant difference between the same Pi incubation groups with different deletion regions.

To investigate the functional regulatory regions and the Pi-induced response of *slc20* promoters, we performed the 5′deletion assays. For grass carp *slc20a1a* promoter, the −1813/−1355 bp sequence deletion did not markedly affect its relative luciferase activity, but the following deletion to −795 bp and the −795/−263 bp increased the luciferase activity significantly (Figure 2A), indicating that the −1355/−263 bp region negatively controlled its promoter activity.

For the *slc20a1b* promoter, the−1804/−1374 bp sequence deletion did not influence the relative luciferase activity, but the subsequent deletion to −899 bp significantly increased the luciferase activity, indicating that the −1374/−899 bp region negatively controlled its promoter activity. However, the −899/−383 bp sequence deletion significantly reduced the luciferase activity, indicating that the −899/−383 bp region positively regulated its promoter activity (Figure 2B).

For the *slc20a2* promoter, the −1775/−1296 bp sequence deletion significantly increased its relative luciferase activity. The subsequent deletion to −835 bp did not influence its relative luciferase activity but the −835/−191 bp sequence deletion significantly increased the luciferase activity, indicating that the −1775/−1296 bp and −835/−191 bp region positively regulated its promoter activity. (Figure 2C).

Compared with the control, Pi incubation significantly reduced the luciferase activities of pGl3-1813/+226, pGl3-1355/+226, pGl3-795/+226 and pGl3-263/+226 of *slc20a1a* plasmids (Figure 2D), the luciferase activities of pGl3-1804/+143, pGl3-1374/+143, and pGl3-899/+143 of *slc20a1b* plasmids (Figure 2E), and the luciferase activities of pGl3-1775/+221 and pGl3-1296/+211 of *slc20a2* plasmids (Figure 2F).

### 2.4. Site-Mutation Analysis of SREBP1, NRF2 and VDR on the Promoters of Slc20s

**Figure 3:**
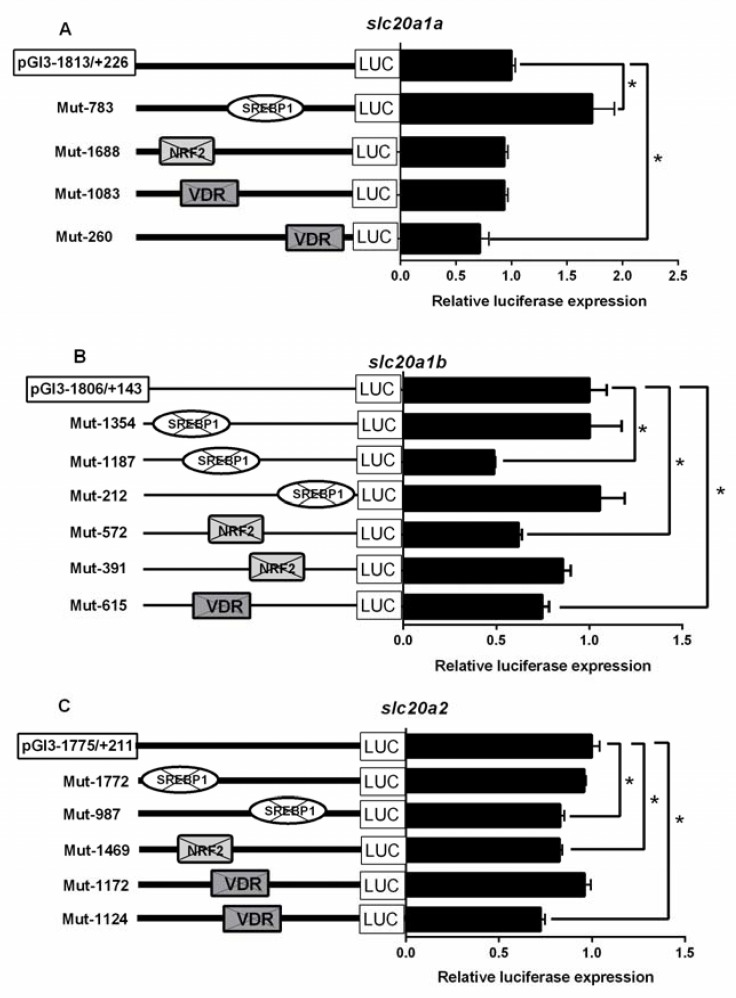
Analysis of putative transcript factor binding sites by site-directed mutagenesis. (**A**) Mutations of *slc20a1a* promoter at −783/−771, −1688/−1679, −1083/−1076, and −260/−253 sites; (**B**) Mutations of *slc20a1b* promoter at −1354/−1345, −1187/−1178, −212/−203, −572/−561, −391/380, and −615/−609 sites; (**C**) Mutations of *slc20a1b* promoter at −1772/−1763, −987/−977, −1469/−1459, −1172/−1165, and −1124/−1117 sites; values represent the ratio between firefly and Renilla luciferase activities. Results are expressed as the mean ± SEM arbitrary units of three independent experiments. Student’s t-test was undertaken to detect the differences between two groups. Symbol (*) indicates significant differences between the two groups.

In order to identify the roles of SREBP1, NRF2 and VDR binding sites on the promoter regions of *slc20s* in grass carp, we performed the site-directed mutagenesis. Compared to the wild-type *slc20a1a* vector, the mutation of SREBP1 binding site (−783/−771 bp) increased but the mutation of VDR (−260/−253 bp) binding site reduced the luciferase activities of *slc20a1a* promoter, indicating that the SREBP1 (−783/−771 bp) negatively but VDR (−260/−253 bp) binding site positively regulated *slc20a1a* promoter activities. However, the mutation of NRF2 (−1688/−1679 bp) and VDR sites (−1083/−1076 bp) did not influence the luciferase activities of *slc20a1a* promoter (Figure 3A).

Compared with the wild-type *slc20a1b* vector, the mutation of SREBP1 (−1187/−1178 bp), NRF2 (−572/−561 bp) and VDR (−615/−609 bp) binding sites significantly decreased the luciferase activities of *slc20a1b* promoter, implying that the SREBP1 (−1187/−1178 bp), NRF2 (−572/−561 bp) and VDR (−615/−609 bp) binding sites positively regulated *slc20a1b* promoter activities. However, the mutation of SREBP1 (−1354/−1345 bp and −212/−203 bp) and NRF2 (−391/380 bp) binding sites did not affect the luciferase activities of *slc20a1b* promoter (Figure 3B).

Compared with the wild-type *slc20a2* vectors, the mutation of SREBP1 (−987/−977 bp), NRF2 (−1469/−1459 bp) and VDR (−1124/−1117 bp) binding sites decreased the activities of *slc20a2* promoter, suggesting that the SREBP1 (−987/−977 bp), NRF2 (−1469/−1459 bp) and VDR (−1124/−1117 bp) binding sites positively regulated *slc20a2* promoter activities. However, the mutations of SREBP1 (−1772/−1763 bp) and VDR (−1172/−1165 bp) binding sites did not affect the luciferase activities of *slc20a2* promoter (Figure 3C).

### 2.5. EMSA Analysis of SREBP1, NRF2 and VDR Binding with the Slc20s Promoters

Next, we examined whether SREBP1, NRF2 and VDR functionally bind with their corresponding regions of *slc20s* promoters using EMSA analysis.

**Figure 4:**
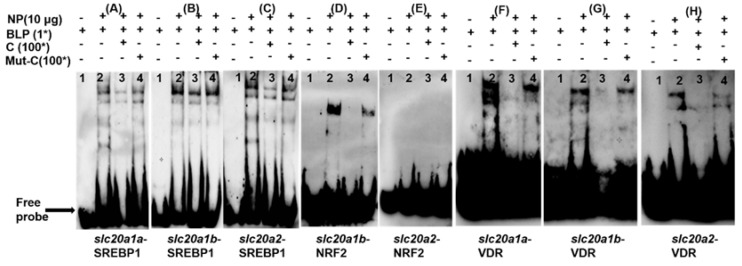
EMSA of putative transcription factors binding sequence. The 5′-biotin labeled double-stranded oligomers were incubated with HEK293T nuclear extract. A 100-fold excess of the competitor and mutative competitor oligomers was added to the competition and mutant competition assay, respectively. NP represents nuclear protein; BLP represents biotin-labeled-probe; C represents competitor oligomers; Mut-C represents mutative competitor oligomers. The oligonucleotide sequences are given in Table S4. (**A**) SREBP1 binding sequences located at −783/−771 bp of the *slc20a1a* promoter. (**B**) SREBP1 binding sequences located at −1187/−1178 bp of the *slc20a1b* promoter. (**C**) SREBP1 binding sequences located at −987/−977 bp of the *slc20a2* promoter. (**D**) NRF2 binding sequences located at −572/−561 bp of the *slc20a1b* promoter. (**E**) NRF2 binding sequences located at −1469/−1459 bp of *slc20a2* promoter. (**F**) VDR binding sequences located at 260/−253 bp of the *slc20a1a* promoter. (**G**) VDR binding sequences located at −615/−609 bp of the *slc20a1b* promoter. (**H**) VDR binding sequences located at −1124/−1117 bp of the *slc20a2* promoter.

For the SREBP1 binding assay, the 100-fold unlabeled SREBP1 binding sequence competed for the binding when we used biotin-labeled SREBP1 binding sequences (−783/−771 bp of *slc20a1a* promoter) as the probe, while the 100-fold unlabeled mutated SREBP1 binding sequences markedly reduced this competition, indicating that the SREBP1 binding sequence (−783/−771 bp of *slc20a1a* promoter) was functionally bound by SREBP1 (Figure 4A). The SREBP1 binding elements at −1187/−1178 bp of the *slc20a1b* promoter (Figure 4B) and at −987/−977 bp of the *slc20a2* promoter (Figure 4C) were functionally bound by SREBP1.

For the NRF2 binding assay, the 100-fold unlabeled NRF2 binding sequence competed for the binding when we used biotin-labeled NRF2 binding sequence (−572/−561 bp of *slc20a1b* promoter) as the probe, while the 100-fold unlabeled mutated NRF2 binding sequence markedly reduced this competition, indicating that NRF2 binding sequence (−572/−561 bp of *slc20a1b* promoter) was bound by NRF2 (Figure 4D). However, no band was found when we used NRF2 binding sequence (−1469/−1459 bp of *slc20a2* promoter) as the probe, suggesting that this sequence was not bound by any factors (Figure 4E).

For the VDR binding assay, the 100-fold unlabeled VDR binding sequence competed for the binding when we used biotin-labelled VDR binding sequence (−260/−253 bp of *slc20a1a* promoter) as the probe, while the 100-fold unlabeled mutated VDR binding sequence markedly reduced this competition, indicating that VDR binding sequence (−260/−253 bp of *slc20a1a* promoter) was bound by VDR (Figure 4F). The VDR binding elements located at −615/−609 bp of *slc20a1b* promoter (Figure 4G) and at −1124/−1117 bp of the *slc20a2* promoter (Figure 4H) were functionally bound by VDR.

### 2.6. Effect of Pi Incubation on the Luciferase Activity of the Site-Mutagenesis Plasmids

**Figure 5:**
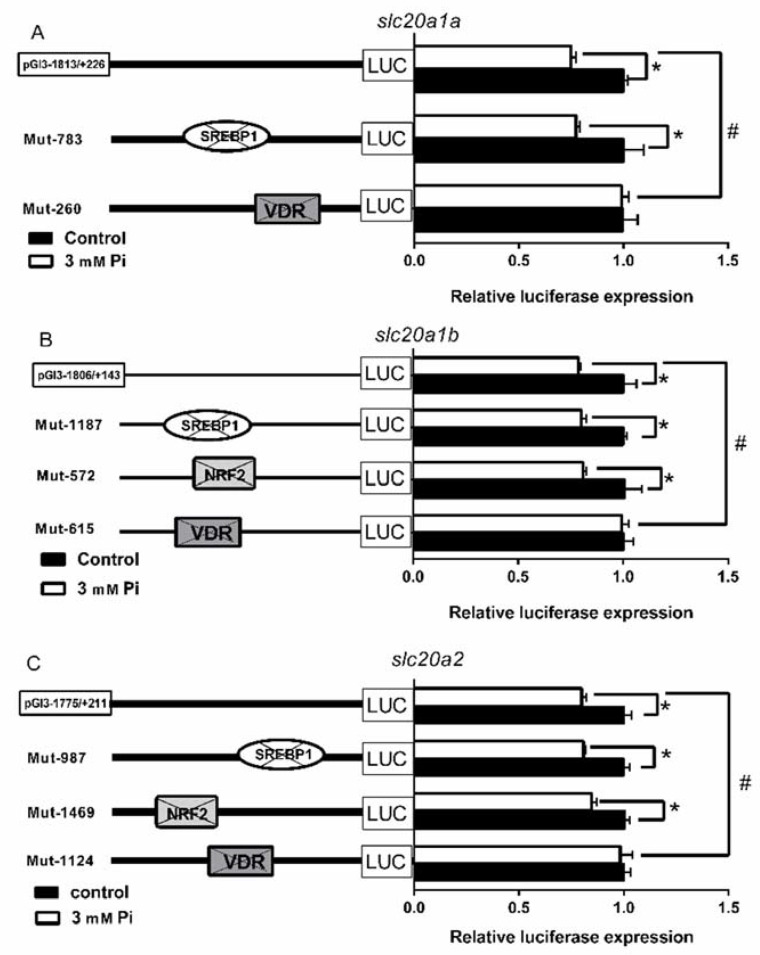
(**A**) Effect of Pi incubation on the promoter activities of *slc20a1a* after SREBP1 and VDR site mutagenesis. (**B**) Effect of Pi incubation on the promoter activities of *slc20a1b* after SREBP1, NRF2 and VDR site mutagenesis. (**C**) Effect of Pi incubation on the promoter activities of *slc20a2* after SREBP1, NRF2 and VDR site mutagenesis. Student’s t-test was undertaken to detect the differences between two treatments. Asterisk (*) indicates significant differences between control and Pi treatment group in the same plasmid (*p* < 0.05). Hash symbol (#) indicates significant differences between different 5′ unidirectional deletion plasmids under the same treatment (*p* < 0.05).

To further determine whether Pi-induced decrease of *slc20s* promoter activities could be mediated by SREBP1, NRF2 and VDR elements, we conducted the site-directed mutation at their corresponding regions of *slc20s* promoters and used Pi to incubate the cells. Pi incubation significantly decreased three *slc20s* promoter activities. However, compared to the wild type pGl3-1813/+226 vector of *slc20a1a*, Pi-induced transcriptional inactivation of *slc20a1a* was abolished after the mutation of VDR element but not SREBP1 element (Figure 5A). Compared with the wild-type pGl3-1801/+143 vector of *slc20a1b*, Pi-induced transcriptional inactivation of *slc20a1b* was abolished after the mutation of VDR element but not SREBP1 and NRF2 elements (Figure 5B). Similarly, compared with the wild-type pGl3-1775/+211 vector of *slc20a2*, the Pi-induced transcriptional inactivation of *slc20a2* was abolished after the mutation of VDR element but not the mutation of SREBP1 and NRF2 elements (Figure 5C).

### 2.7. Effect of Pi Incubation on the mRNA Level of Three slc20s Gene in CIK Cells of Grass Carp

**Figure 6:**
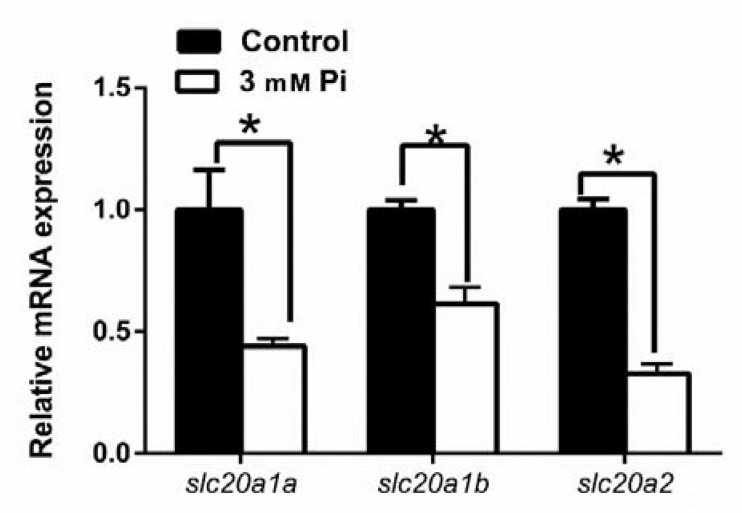
Effect of Pi incubation on the mRNA level of *slc20s* in CIK cells. Student’s t-test was undertaken to detect the differences between two treatments. Asterisk (*) indicates significant differences between control and Pi treatment group (*p* < 0.05).

To identify whether the expressions of *slc20s* were regulated by Pi in grass carp, we cultured CIK cells and used Pi to incubate the cells. Compared to the control, 3 mM Pi incubation markedly reduced the mRNA abundances of three *slc20s* genes (Figure 6).

## 3. Discussion

In mammals, the SLC20 family includes two members (SLC20A1 and SLC20A2), which are encode by *slc20a1* and *slc20a2* genes [13]. In contrast, the present study obtained three members of the SLC20 family (*slc20a1a*, *slc20a1b* and *slc20a2*) from grass carp. Thus, grass carp show more *slc20a1* isoforms than mammals, which is attributable to the TSGD events [22]. Moreover, our study indicated that grass carp SLC20A1A, SLC20A1B and SLC20A2 possessed 11, 11 and 10 TMDs, respectively (Figure S2). Similarly, 10–12 TMDs of SLC20s were also predicted in mammals [23,24]. Many amino acid residues important for Pi transport function in human, such as D28, E55, D506 and E575, were also conserved in the SLC20A2 from grass carp [25,26,27]. Additionally, Virkki et al. [25] suggested a conserved N-glycosylated site Asp−81, which was also found in the Asp−81 in grass carp of SLC20A2.

It is very important to explore the mRNA tissue expression of genes, which provides the knowledge into their potential functions. The present study indicated that the *slc20a1a*, *slc20a1b* and *slc20a2* mRNA amounts were ubiquitously existent in tissues but varied (Figure 1), in consistence with other reports [9,28,29]. As a matter of fact, due to their broad distribution, they are considered to act as “housekeeping” roles for Pi transport into the cells [2,25]. Moreover, we found that *slc20a1a* mRNA amounts were the highest in the kidney and liver and the *slc20a1b* mRNA expression was predominant in the kidney, and that the *slc20a2* mRNA amounts were predominant in the brain, suggesting their importance in these tissues. Similarly, Beck et al. [30] reported that *slc20a1* was a critical gene for the normal growth and development of the liver. The predominant *slc20a1a* and *slc20a1b* mRNA expression in the kidney suggested that they shared the similar function in Pi reabsorption in the tissue. For *slc20a2*, Inden et al. [5] pointed out that the mRNA levels of *slc20a2* were higher in the brain than those in the liver and kidney in mammals. Similarly, Jensen et al. [31] indicated that *slc20a2* played important roles in keeping Pi homeostasis in the calcification of the brain.

In eukaryotes, in order to explore the mechanism of transcriptional initiation of genes, the first step is to identify their core promoter regions [32]. In the present study, three *slc20s* genes had different structures of the core promoters. For example, the core promoters of *slc20a1a* had both CCAAT-box (−39 bp) and TATA-box (−12 bp), the core promoters of *slc20a1b* only had TATA-box (−14 bp), but the core promoters of *slc20a2* had CCAAT-box (−93 bp) and Sp1 sites (−72 bp, −10 bp), indicating that three *slc20s* had different regulatory mechanism for their transcriptional initiation (Figure S1). Usually, TATA-less promoters possessed various Sp1 binding sites in their promoter regions [33]. In order to decipher the transcriptionally regulatory mechanisms, it is very important to identify the TFBSs of gene promoters [34]. SREBP1 is an important transcriptional factor that regulates the genes involved in lipogenesis [35]. Several reports suggested that Pi influenced lipid deposition [36,37] and that SREBP1 increased lipogenesis and accelerated vascular calcification in mammals [15,16]. Interestingly, SLC20s also play key roles in cell calcification in mammals [5,38,39]. Our study identified the functional SREBP1 binding sites on the *slc20s* promoters (Figure 4A–C). We speculated that SREBP1 mediated the transcriptional regulation of SLC20s expression. Moreover, we found that SREBP1 positively regulated the activities of *slc20a1b* and *slc20a2* promoters, and negatively regulated the promoter activity of *slc20a1a* (Figure 3A), suggesting that SREBP1 differentially regulated Pi transport by targeting different *slc20s*. Our study also indicated the differentially transcriptional regulatory mechanism for duplicated genes *slc20a1a* and *slc20a1b*. Studies point out that duplicated genes will result in neofunctionalization and subfunctionalization [40,41]. NRF2 is a crucial transcription factor that influences intrinsic resistance to oxidative stress [42]. Pi regulates the production of reactive oxygen species (ROS) [43]. Phosphate load increased oxidative stress and led to tissue damages and cytoxicity in various models [44,45]. In this study, we found that NRF2 positively regulated the promoter activity of *slc20a1b*, but not *slc20a1a* and *slc20a2* (Figure 3B and Figure 4D,E), indicating that *slc20a1b* might play important role than *slc20a1a* and *slc20a2* under oxidative stress. Michigami et al. [13] suggested that NRF2 mediated the direct influences on extracellular Pi transport. Wei et al. [46] found that the activation of NRF2 signaling alleviated high phosphate-induced calcification of vascular smooth muscle cells by suppressing the ROS production. Moreover, several studies also suggested that NRF2 was involved in osteogenic differentiation and mineralization [47,48]. Considering that the important functions of SLC20 in osteogenic differentiation and cell calcification, we speculated that NRF2 probably regulated osteogenic differentiation and cell calcification by targeting *slc20a1*. The VDR is a zinc-finger transcription factor regulating the expression of several genes and important for the regulation of Pi homeostasis [12]. Our site-mutation and EMSA analysis identified the functional VDR binding sequences on *slc20a1a*, *slc20a1b* and *slc20a2* promoters (Figure 4F–H), and found that the mutation of VDR decreased the luciferase activities of three *slc20* promoters (Figure 3). Taken together, it seems that three *slc20* genes possessed not only the similar but also some different transcriptional regulatory mechanism.

Although *slc20a1* and *slc20a2* are expressed in various tissues, evidence was scarce about whether these *slc20* transporters controlled the phosphate homeostasis at the cellular levels. Using cell lines from mammals, studies found that *slc20a1* and *slc20a2* mediated Pi transport and their effects are regulated by Pi levels [49]. In the present study, we found that 3 mM Pi incubation markedly reduced the mRNA abundances of three *slc20s* (Figure 6) and decreased the activities of three *slc20s* promoters (Figure 2). Similarly, Chien et al. [50,51] reported that phosphate starvation increased *slc20a1* and *slc20a2* gene expression and induced the posttranslational modifications of *slc20a2* that accordingly activated phosphate transport. Other studies indicated that *slc20a1* and *slc20a2* had redundant roles in the regulation of Pi transport. For example, in mouse VSMCs, Crouthamel et al. [52] found that the *slc20a2* knockdown in the *slc20a1*-deficient mouse VSMCs decreased Pi uptake, but the deletion of the *slc20a1* gene alone had no effects on Pi uptake, probably due to the compensatory upregulation of *slc20a2*. In contrast, other studies indicated different functions in the two SLC20 transporters. For example, Villa-Bellosta and Sorribas [53] found that SLC20A1, not SLC20A2, was the only Pi transporter in the kidney that was not up-regulated in response to dietary Pi deprivation. Thus, it would be important to clarify the redundancy of SLC20A1 and SLC20A2 in Pi transport. Moreover, we found that VDR, but not SREBP1 and NRF2, mediated Pi-induced transcriptional inactivation of *slc20a1a*, *slc20a1b* and *slc20a2* (Figure 5), suggesting that VDR binding sites directly mediated the *slc20s* transcriptional response to Pi. Similarly, other studies indicated that *slc20a1* and *slc20a2* expression can be positively regulated by VDR [9,12]. Although SREBP1 and NRF2 did not directly mediate Pi-induced transcriptional inactivation of *slc20s*, they may be mediated by some other factors. For example, Kawamoto et al. [54] reported that high-fat diets increased the expression of *slc20a1* and provoke phosphorus absorption in rats, which may directly mediated by SREBP1.

## 4. Materials and Methods

### 4.1. Experimental Grass Carp and Reagents

The grass carp juveniles were bought from a farm in Wuhan (China) and transported to our wet laboratory in Huazhong Agricultural University (HZAU) for the culture. CIK and HEK293T cell lines came from the Cell Resource Center in Fishery College of HZAU. All these media and reagents for cell cultures were purchased from Gibco company (ThermoFisher Scientific, Waltham, MA, USA). All these animal experiments followed the guideline of the Animal Experimentation Ethics Committee of HZAU (Wuhan, Hubei, China) and were approved by Ethics Committee of HZAU (Fish-2019-0418, 18 April 2019).

### 4.2. Sequence Clone and Plasmids Construction

Total RNA and genomic DNA were extracted from nine tissues of grass carp (heart, brain, intestine, kidney, liver, muscle, gill, spleen and skin) by using TRIzol RNAiso reagent (Invitrogen, Carlsbad, CA, USA) and commercial DNA extracted kit (Omega, Norcross, GA, USA). Total RNA was then reverse-transcribed to cDNA by using the oligo-dT primers and commercial cDNA Synthesis Kits (TaKaRa, Otsu, Japan). Core cDNA and promoter sequences of *slc20a1a*, *slc20a1b* and *slc20a2* were obtained by RT-PCR, with the reference of the genome of grass carp [55]. The 3′- and 5′-end sequences were cloned via the nested 3′- and 5′-RACE PCR. Table S1 showed all these primers.

In order to generate the luciferase reporter constructs, we used Sac I and Hind III sites as the restriction sites of PCR products and pGl3-Basic vectors. They were purified and digested using corresponding endonucleases. Then, we ligated the products via the One-Step Cloning Kit (Vazyme, Piscataway, NJ, USA). According to the distance from their transcription start sites (TSS), we named these plasmids as the vectors of pGl3-1813/+226 of *slc20a1a*, pGl3-1804/+143 of *slc20a1b* and pGl3-1775/+221 of *slc20a2*, respectively. Then, we used the templates of pGl3-1813/+226 of *slc20a1a* vector to produce the plasmids pGl3-1355/+226, pGl3-795/+226, and pGl3-263/+226 of *slc20a1a* vectors. Similarly, the plasmids pGl3-1374/+143, pGl3-899/+143, and pGl3-383/+143 of *slc20a1b* vector, pGl3-1296/+211, pGl3-835/+211 and pGl3-191/+211 of *slc20a2* vector were produced by using the pGl3-1804/+143 of *slc20a1b* vector and pGl3-1775/+221 of *slc20a2* vector as the templates, respectively. We performed the PCR via the TaKaRa PrimeSTAR^®^ HS DNA Polymerase kit (TaKaRa, Tokyo, Japan). Finally, we sequenced all these plasmids in the Tsingke company (Wuhan, China). The primers for the plasmid construction were presented in Table S2.

### 4.3. Sequence Analysis

We used BLAST network service at the NCBI (http://blast.ncbi.nlm.nih.gov/) to compare the nucleotide sequences with DNA sequences from the GenBank database. TMpred (http://www.ch.embnet.org/software/TMPRED_form.html) was utilized to predict the putative transmembrane regions, and the Clustal-W multiple alignment algorithm for analyzing the sequence alignments and percentage of amino acid conservation. Several online software programmes, such as MatInspector (http://www.genomatix.de/), the JASPAR database (http://jaspar.genereg.net/) and the TFSEARCH database (http://www.cbrc.jp/research/db/TFSEARCH.html), were utilized to analyze the potential transcription factor binding sites (TFBS).

### 4.4. Plasmid Transfections and Analysis of Luciferase Activities

HEK293T cells were cultured in DMEM medium with 10% fetal bovine serum (FBS) (Gibco, Carlsbad, CA, USA) in an incubator (5% CO_2_ and 37 °C). Prior to the transfection, HEK293T cells were seeded at a density of 1.2 × 10^5^ in 24-well plate. They were cultured for 24 h to reach 70–80% confluence. Lipofectamine™2000 (Invitrogen, Carlsbad, CA, USA) was utilized to transfect all these plasmids into HEK293T cells, based on the manufacture’s protocol. The 400-ng reporter plasmids were co-transfected with 20 ng pRL-TK, the internal control with a Renilla luciferase reporter vector. HEK293T cells were incubated for 24 h and collected to analyze the luciferase activity (Dual-Luciferase Reporter Assay System, Promega, San Luis Obispo, CA, USA). For an internal control, we added 20 ng of *Renilla* luciferase vector (pRL-TK) per well to normalize the efficiency of the transfection. After 4h, we replaced the transfection medium by 10% FBS-DMEM or 10% FBS-DMEM + 3 mM Pi (Pi was added as a mixture of NaH_2_PO_4_ and Na_2_HPO_4_, pH7.4). The form and the concentration of Pi was selected according to our pilot trial and to previous in vitro studies [56,57,58,59]. Then, after 24-h incubation, cells were collected to determine the promoter activity, based on the manufacturer’s instruction of the Dual-luciferase Reporter Assay System (Promega, San Luis Obispo, CA, USA). The relative luciferase activities were obtained by calculating the ratio of *Firefly* luciferase activity to *Renilla* luciferase activity. We conducted all these experiments in triplicates.

### 4.5. Site-Mutation Analysis of SREBP1, NRF2 and VDR Binding Sites on the Regions of Three Slc20s Promoters

In order to characterize the SREBP1, NRF2 and VDR binding sites on the *slc20a1a*, *slc20a1b* and *slc20a2* promoters of grass carp, we used QuickChange II Site-Directed Mutagenesis Kit (Vazyme, Piscataway, NJ, US) to undertake the site-directed mutagenesis, and pGl3-1813/+226 of the *slc20a1a* vector, pGl3-1804/+143 of the *slc20a1b* vector and pGl3-1775/+221 of the *slc20a2* vector as the templates, respectively. Several mutations were performed, as shown below: (1) at the sites of −783/−771 bp, −1688/−1679 bp, −1083/−1076 bp and −260/−253 bp sites for the *slc20a1a* promoter; (2) at the sites of −1354/−1345 bp, −1187/−1178 bp, −212/−203 bp, −572/−561 bp, −391/380 bp and −615/−609 bp sites for the *slc20a1b* promoter; (3) at the sites of −1772/−1763 bp, −987/−977 bp, −1469/−1459 bp, −1172/−1165 bp and −1124/−1117 bp sites for the *slc20a2* promoter. The primers used for mutagenesis were shown in Table S3. The DNA sequencing was utilized to confirm these mutations. Then, the Lipofectamine 2000 reagent (Invitrogen, Carlsbad, CA, USA) was utilized to co-transfect these plasmids and pRL-TK into HEK293T cells. After 4-h transfection, the medium was substituted with 10% FBS-DMEM or 10% FBS-DMEM + 3 mM Pi (Pi was added as a mixture of NaH_2_PO_4_ and Na_2_HPO_4_, pH7.4). After 24-h incubation, we harvested the cells to determine the luciferase activities, based on the procedures mentioned above.

### 4.6. Electrophoretic Mobility-Shift Assay (EMSA)

The EMSA was undertaken to confirm the functional SREBP1, NRF2 and VDR binding sites on the three slc20s promoters according to our recent publications [60,61,62]. Nuclear and cytoplasmic extracts were prepared for after the methods of Read et al. [63]. Protein contents were determined by the BCA method [64]. The oligonucleotide probes were synthesized in the Tsingke company (Wuhan, China). Nuclear extracts (10 µg) were incubated for 30 min at the room temperature by using the binding buffer (20 mM HEPES, pH7.9, 1 mM MgCl_2_, 0.5 mM DTT, 4% Ficoll, 110 mM KCl, 0.2 μg Poly(dI-dC)). Then, the biotin-labeled double-stranded oligo nucleotides (Table S4) were added. The reaction continued for 30 min and then the electrophoresis was performed on 6% native polyacrylamide gels. For the competitive binding analysis, a 100-fold excess of unlabeled double-stranded DNA oligo with mutant binding site (Table S4) was added with the corresponding labeled one.

### 4.7. Effect of Pi Incubation on the mRNA Level of Slc20s in CIK Cells of Grass Carp

We cultured grass carp CIK cells in DMEM medium plus 10% FBS (Gibco, Carlsbad, CA, USA) in a SANYO incubator (28 °C and 5% CO_2_). The CIK cells were seeded at a density of 1.2 × 10^5^ in the 6-well plate. They were cultured until the 70–80% confluence was reached. Then we replaced the medium by 10% FBS-DMEM or 10% FBS-DMEM + 3 mM Pi (Pi was added as a mixture of NaH_2_PO_4_ and Na_2_HPO_4_, pH7.4). After 24 h, we collected the CIK cells. The total RNAs were isolated from these CIK cells and reverse-transcribed to cDNA as the templates.

### 4.8. mRNA Expression after Quantitative Real-Time PCR (Q-PCR) Analysis

The *slc20s* mRNA levels in nine tissues and CIK cells were determined by Q-PCR analysis, based on our published protocols [60,61]. All of these primers are given in Table S5. Q-PCR assays were undertaken in a quantitative thermal cycler (MyiQ™ 2 Two Color Quantitative PCR Detection System, BIO-RAD, USA). Five housekeeping genes (*β-actin*, *18s-rrna*, *b2m*, *gapdh* and *ef-1α*) were selected to analyze their transcription stability. Two most stable control genes (*gapdh* and *18s-rrna*) were selected by geNorm software analysis [65]. The mRNA expression levels were calculated via the “delta–delta Ct” method [66].

### 4.9. Statistical Analysis

We showed the results with the means ± standard error of mean (SEM) at three independent experiments. Prior to statistical analysis, all data were tested for normality of distribution using the Kolmogornov–Smirnov test. The homogeneity of variances was tested using Barlett’s test. For the *slc20* tissue mRNA expression patterns analysis, the data were subjected to one-way ANOVA and Tukey’s multiple range test. For assay of promoters’ activities, Student’s *t*-test was undertaken to detect the differences between two treatments. The significant level was set at *p* < 0.05. All of these data were analyzed by using the SPSS 19.0 software (SPSS Inc., Chicago, IL, USA).

## 5. Conclusions

For the first time, the full cDNA lengths and the promoters of three slc20 members (*slc20a1a*, *slc20a1b* and *slc20a2*) were isolated and characterized from fish. They shared similar TMDs to mammals and their mRNAs were existent among various tissues. Three transcription factors (SREBP1, NRF2 and VDR) regulated the transcription activities of three *slc20s* promoters and the transcriptional inactivations of *slc20s* were mediated by VDR under Pi treatment. Our study partially elucidated the mechanism of Pi homeostatic regulation of vertebrates and provided a good basis for dietary Pi addition and for the reduction of Pi discharge into the waters.

## Figures and Tables

**Table 1:** The sequences information of three *slc20s* from grass carp *C. idella.*

Genes/Name	Promoters/bp	5‘UTR/bp	ORF/bp	3’UTR/bp	Protein/bp	Full-Length/bp
*slc20a1a*	1813	180	1935	377	644	2264
*slc20a1b*	1804	76	1995	410	664	2453
*slc20a2*	1775	140	1989	79	662	2125

**Table 2:** Amino acid sequence identities of SLC20s between grass carp *C. Idella* *(Ci)* and other species (%).

Genes	*Ci*-*slc20a1b*	*Ci*-*slc20a2*	*Danio rerio*	*Cyprinus carpio*	*Xenopus*	*Canis lupus*	*Mus musculus*	*Homo sapiens*
*Ci*-*slc20a1a*	*73*	*61.9*	92.4	94.7	67.4	67.0	66.9	67.7
*Ci*-*slc20a1b*	*_*	*60.0*	91.7	90.9	71.4	70.2	69.8	71.3
*Ci*-*slc20a2*	_	_	95.5	94.4	79.0	76.4	76.6	78.1

Note: *slc20a1a* protein ID of *Danio rerio*, *Cyprinus carpio*, *Xenopus*, *Canis lupus*, *Mus musculus*, and *Homo sapiens* are as follows: ENSDART00000010993.10, ENSCCRT00015001389.1, NP_001087494.1, XP_540181.2, NP_056562.1, and NP_005406.3; *slc20a1b* protein ID of *Danio rerio*, *Cyprinus carpio*, *Xenopus*, *Canis lupus*, *Mus musculus*, and *Homo sapiens* are as follows: NP_997753.1, ENSCCRT00015024323.1, NP_001083287.1, XP_540181.2, NP_056562.1, NP_005406.3; *slc20a2* protein ID of *Danio rerio*, *Cyprinus carpio*, *Xenopus*, *Canis lupus*, *Mus musculus*, and *Homo sapiens*: ENSDART00000149297.3, ENSCCRT00015021589.1, AAH84098.1, XP_005629854.1, NP_035524.2, and NP_001244109.1.

## Supplementary Materials

Supplementary Materials can be found at https://www.mdpi.com/1422-0067/21/21/8228/s1.

## Abbreviations

*18srrna*	18SrRNA
EMSA	electrophoretic mobility-shift assay
*gapdh*	Glyceraldehyde-3-phosphate dehydrogenase
MS-222	Tricaine methanesulfonate
NRF2	Nuclear factor-erythroid 2 p45-related factor 2
ORF	Open-reading frame
Pi	Phosphorus
RACE	rapid amplification of cDNA ends
SREBP1	Sterol regulatory element-binding protein
TFBS	transcription factor binding site
TSS	transcription start sites
UTR	Untranslated region
VDR	Vitamin D receptor
