# Supplementary material for: Isolation and Characterization of Three Sodium-Phosphate Cotransporter Genes and Their Transcriptional Regulation in the Grass Carp Ctenopharyngodon idella
Source: Int J Mol Sci. 2020 Nov 3;21(21):8228. doi: 10.3390/ijms21218228 (PMC7662828; doi:10.3390/ijms21218228)
Supplement: Supplementary file 1 [file ijms-21-08228-s001.pdf]

Table S1. Primers used for *slc20a1a, slc20a1b,* and *slc20a2* gene cloning.

Gene	Forward primer (5′-3′)	Reverse primer (5′-3′)	Step
slc20a1a	CTAGCATCCCTTGCGACTG	CAGAAATTGGCACGGTCA	Partial
	TCATCCTGTAAGTTCTCCAGCATC	GAACTTGCGGACAAAGTAGAA	5′ outer
	CGCGGATCCACAGCCTACTGATGATCAGTCGATG	ACTGTCTCAAAGATGGTGGC	5′inner
	ATGGGCAAAGACCTGACTC	TACCGTCGTTCCACTAGTGATTT	3′outer
	GTGCCAATTTCTGGTCTCAT	CGCGGATCCTCCACTAGTGATTTCACTATAGG	3′inner
	TAACAGGCAAGACTGATCTAGAGT	CATGGTAACAGTCGCAAGGGATG	promoter
slc20a1b	GCCACGATAACGCTTTTG	CCAAGCCATGAAGATGTTTC	Partial
	TCATCCTGTAAGTTCTCCAGCATC	ACATGCTCTGAGCCGTTGTA	5′ outer
	CGCGGATCCACAGCCTACTGATGATCAGTCGATG	AAGAATACAAGCCTGCCTAAGAG	5′inner
	TGGGTCAGTGGTGTCAGTCG	TACCGTCGTTCCACTAGTGATTT	3′outer
	GAAACATCTTCATGGCTTGGT	CGCGGATCCTCCACTAGTGATTTCACTATAGG	3′inner
	GGTTTACTGTTTGACGCCACCAT	TGTATTGATTGCAGCATACCCTG	promoter
slc20a2	CTTCATTATAGCCTTCATCCTGG	GCATTATGCCGTAGACGAACA	Partial
	TCATCCTGTAAGTTCTCCAGCATC	GCCAAACCGCAGACCCAACC	5′ outer
	CGCGGATCCACAGCCTACTGATGATCAGTCGATG	TGGAGCCCAGGGTCTCAAAT	5′inner
	GGCAAAGACCTCACTCCAATC	TACCGTCGTTCCACTAGTGATTT	3′outer
	CTCTAAGAAGGCTGTGGACTGG	CGCGGATCCTCCACTAGTGATTTCACTATAGG	3′inner
	GGTCTGTGGTGAGAACGCTCTGT	CACTGAAAATGCCAGGATGAAGG	promoter

Table S2. Primers used for 5′-deletion plasmids construction.

Primers	Forward primer (5′-3′)	Reverse primer (5′-3′)
slc20a1a		
pGl-1813/+226	ctatcgataggtaccgagctcTAACAGG CAAGACTGATCTAGAGT	cagtaccggaatgccaagcttCATGGTAAC AGTCGCAAGGGATG
pGl-1355/+226	ctatcgataggtaccgagctcGTGTATGT ATTCTTGTGTGGTCAGG	cagtaccggaatgccaagcttCATGGTAAC AGTCGCAAGGGATG
pGl-795/+226	ctatcgataggtaccgagctcTGTCTTTG TTGTTTACCACCCCA	cagtaccggaatgccaagcttCATGGTAAC AGTCGCAAGGGATG
pGl-263/+226	ctatcgataggtaccgagctcCTATTTTG CACCCACAAATCACCATT	cagtaccggaatgccaagcttCATGGTAAC AGTCGCAAGGGATG
slc20a1b		
pGl-1804/+143	ctatcgataggtaccgagctcGGTTTAC TGTTTGACGCCACCAT	cagtaccggaatgccaagcttTGTATTGATT GCAGCATACCCTG
pGl-1374/+143	ctatcgataggtaccgagctcTTATTTGT GAACGGCTAGAATGAGC	cagtaccggaatgccaagcttTGTATTGATT GCAGCATACCCTG
pGl-899/+143	ctatcgataggtaccgagctcACAGCCC TACAGTGACAGACAGT	cagtaccggaatgccaagcttTGTATTGATT GCAGCATACCCTG
pGl-383/+143	ctatcgataggtaccgagctcCTCTGTCT GCCTAATTGCCCAAG	cagtaccggaatgccaagcttTGTATTGATT GCAGCATACCCTG
slc20a2		
pGl-1775/+211	ctatcgataggtaccgagctcGGTCTGT GGTGAGAACGCTCTGT	cagtaccggaatgccaagcttCACTGAAAA TGCCAGGATGAAGG
pGl-1296/+211	ctatcgataggtaccgagctcGCTGTTA CATTCTGTTATGGACG	cagtaccggaatgccaagcttCACTGAAAA TGCCAGGATGAAGG
pGl-835/+211	ctatcgataggtaccgagctcCAAACCT GTTACGCATTCTGGCA	cagtaccggaatgccaagcttCACTGAAAA TGCCAGGATGAAGG
pGl-191/+211	ctatcgataggtaccgagctcTTACGCA GTCTATAAGGAAAGTGTG	cagtaccggaatgccaagcttCACTGAAAA TGCCAGGATGAAGG

Table S3. Primers used for site-mutation analysis.

Primers	Forward primer (5′-3′)	Reverse primer (5′-3′)
slc20a1a		
Mut-783	ccgttgttttgatCACGAGATAAAGGTAAAACTACTATATAAAATAG	CGTGatcaaaacaacggACAACAAAGACACGTGGTGTCAA
Mut-1688	cagctggatgGAAGTCATTCAACGCATTTGGTG	ATGACTTCcatccagctgGCTGTGTAAATTGATAAACGCACG
Mut-1083	TTtgaaggtgTAATCAAGTAGCACGCTTGATGAA	CTTGATTAcaccttcaAAAATACTTTTGATATTTTGATGGCAA
Mut-260	TAccccatgCCCACAAATCACCATTCCCCCAA	TTTGTGGGcatggggTAGCCTAACCTAGTCCACATAACTATATAAGC
slc20a1b		
Mut-1354	CGtagatctacaatcccaTCAGCTGACTCGAGGTGGTCG	tgggattgtagatctaCGCTCATTCTAGCCGTTCACA
Mut-1187	gcgctactgaATTAGCTGTTAGCGGGTGTGAAT	CAGCTAATtcagtagcgcAAATCATAAGTACTTAATTAAAACATTCCAA
Mut-212	ATAGGCCtctgtcttgtCCTCTCCCTTAACGTTACACATCA	GacaagacagaGGCCTATTGCTATCCTCTGAATAGA
Mut-572	TAAggacccccaccacgTGATCAGGCATGCATTCATGC	AcgtggtgggggtccTTAAAAATACATATATATGCCATCCGC
Mut-391	TGAAATcaccagacggCACGGGAACCTTCCCACTTT	TGccgtctggtgATTTCACTGCAGAGGGGCCAG
Mut-615	GGTTTcgacctgTGGATAAGCGGATGGCATATATAT	ATCCAcaggtcgAAACCAATGTCTATTTGGCTTTTTG
slc20a2		
Mut-1772	CGGTtcacaacagaAACGCTCTGTAATTTTACAGGTGTTG	CGTTtctgttgtgaACCGAGCTCGGTACCTATCGA
Mut-987	TACggcggtactgcgATATCATCATAATCTTCACATACATATTCTTTT	TATcgcagtaccgccGTATGATTTTAATCGTTGGAAAGAGTCA
Mut-1469	GCTatagtctcatgGTGTTGCACAGTGCAGCTGG	CACcatgagactatAGCCCCTAATACCTGCTTACAGC
Mut-1172	ATATCcagccctgAACCTTGAAATCCCATGTCTTTTG	AGGTTcagggctgGATATTAAATAGCATTACATAAAACAGAAAGA
Mut-1124	CATCTTTATcacgtccgCAAAAAAATATAGCAAATTTTCATAATTT	GcggacgtgATAAAGATGTATTGAACAAAAGACATGG

Table S4. Primers used for electrophoretic mobility-shift assay (EMSA)

Primers	Forward primer (5′-3′)	Reverse primer (5′-3′)
slc20a1a		
B-SREBP1-783	TTTGTTACCACCCCAGCCACGAG	CTCGTGGCTGGGGTGGTAACAAA
M-SREBP1-783	TTTGTCGTTGTTTTGACCACGAG	CTCGTGGTCAAAACAACGACAAA
B-VDR-260	GGCTATTTTGCACCCACAAATCAC	GTGATTTGTGGGTGCAAAATAGCC
M-VDR-260	GGCTACCCCATGCCCACAAATCAC	GTGATTTGTGGGCATGGGGTAGCC
slc20a1b		
B-SREBP1-1187	TATGATTTATAACGTCATATTA	TAATATGACGTTATAAATCATA
M-SREBP1-1187	TATGATTTGCGCTACTGAATTA	TAATTCAGTAGCGCAAATCATA
B-NRF2-572	AAAAGTTTTTGTTGTATGATCA	TGATCATACAACAAAAACTTTT
M-NRF2-572	AAGGACCCCCACCACGTGATCA	TGATCACGTGGTGGGGGTCCTT
B-VDR-615	ATTGGTTTTAGTTCATGGATAAGC	GCTTATCCATGAACTAAAACCAAT
M-VDR-615	ATTGGTTTCGACCTGTGGATAAGC	GCTTATCCACAGGTCGAAACCAAT
slc20a2		
B-SREBP1-987	ATCATACAATAACGTCATAATA	TATTATGACGTTATTGTATGAT
M-SREBP1-987	ATCATACGGCGGTACTGCGATA	TATCGCAGTACCGCCGTATGAT
B-NRF2-1469	TAGGGGCTGCGACTCTGCAGTGTT	ACACTGCAGAGTCGCAGCCCCTA
M-NRF2-1469	TAGGGGCTATAGTCTCATGGTGTT	AACACCATGAGACTATAGCCCCTA
B-VDR-1124	TCTTTATTGTACTTACAAAAAAT	ATTTTTTTGTAAGTACAATAAAGA
M-VDR-1124	TCTTTATCACGTCCGCAAAAAAAT	ATTTTTTTGCGGACGTGATAAAGA

Table S5. Primers used for q-PCR from grass carp

Primers	Forward primer (5′-3′)	Reverse primer (5′-3′)	Accession No.
β-actin	ACCCTGAAGTACCCCATCGA	CAGAGGCATACAGGGACAGC	DQ211096.1
18srrna	GGCGCGCAAATTACCCATT	TCCCGAGATCCAACTACAAGC	EU047719.1
b2m	GCACTCGTCTCTTTTGCCCT	TTTCGAAGGCCAGGTCAGTC	AB128864.1
gapdh	GGGAAACTGTGGAGGGATGG	TGCAGCCTTGACCACTTTCT	GQ245759.1
ef-1α	ACCACCGGCCATCTGATCTAC	GTGTCCAGGGGCATCAATGA	GQ266394.1
slc20a1a	CCATCCGTTCGGGTATCAT	CCACAATGCAGTGCGTTCC	MT561019
slc20a1b	GTAGGCTTCATCATAGCATTT	CCCAGCGTCTCAAAGATAG	MT561020
slc20a2	TAAGAAGGCTGTGGACTGG	CATTATGCCGTAGACGAACA	MT561021

(A)

```
-1813 TAACAGGCAA GACTGATCTA GAGTTAGTTG AGTCGTGGAG TTAAAGTCTA AGAAGGTTTT CGGGGTCATA TTTACTTTGT GTGTTTTACA CAGGAACTGA

-1713 GCGTGCGTTT ATCAATTTAC ACAGCTGATC AAGCAGAAGT CATTCAACGC ATTTGGTGAG TACTCTGTTT AGTTACTTTT AGGTTACATC TGAGAGTTTT
                                 NRF2
-1613 TTTAAGGGTT GCAAAAATTC AGCACTATTT ATTTAACTGT GTATTTGAAC TTGATATGAT TTTAGTAATA TTGGATAGTA ATTTAATACA TTGTTAATTT

-1513 ACATTCATGT CCTTAACAGC AACTCAAAAT AGCATGATGA ACTGCACAAT GTTGGTCATC TTACATCAAT TGTCTTTTCA CAACTATCTG TATTTAATAT
                                        ATF4
-1413 GACCCAGACT TGGCGCTTAC ATAATTAGAA TGTTGACAAA TTCTGATGAT ATTTGTGTAT GTATTCTTGT GTGGTCAGGG AACATCATAA TGATTTGGTA

-1313 GGCTGTTCTT CTGTACAAAG TCAAATCAAA CACCTGTTTT CATAGAATAA TAAGCAATTC AACAAGTCAT TCATTTACTT CTCTTAAGAA GCATGTGCTC

-1213 AAGACTTTGT AAAGTGTTGT AAAGTCTCTA AATCCTCTGA TGTCTGAAGG CACTTATGTA ATAGTCTGTG TTACATCAAA ACTTGCCATC ACAAGGTTAC
                                                                ATF4               ATF4
-1113 GAGTTGCCAT CAAAATATCA AAAGTATTTT CAGGTTCATA ATCAAGTAGC ACGCTTGATG AAGCATTATT AAGCCTCTTT TTTTCCAAAT GAATGTGGCA
                                       VDR                          ATF4
-1013 CTTTGTTTTG GAAATTCCTT ACTTGACTAC TTACACTGGC ACACAGTAAA TCTTAATGTC CCTCAAAGGT TTTTTGGAAA AAACAGTATT ATTTTTATGG
          STAT3                                                        STAT3
-0913 TGGATAAATA ATATGCTATG CTAACAGATT TAAATAGAGA GAAAGAGAGA GAGAGTAAAA AATATGCTTA TAAATAATTA AATGCATTAA AATCACTATC

- 813 CCTTTGTTTG ACACCACGTG TCTTTGTTGT TTACCACCCC AGCCACGAGA TAAAGGTAAA ACTACTATAT AAAATAGTAA GATATTGCTG CAAATATGAA
                                    SREBP1
- 713 ATGTTATCAC TAGCATATTG TGCTTTGCAT TGGTTTTAAA CCAAATTATT ACAGTCTTAG CTGAAAAATG ACTTTATTTT ATTCAAGATT ACTTTATTGG

- 613 TCCCTAAAAA GGCTGCCACA TAAAAACATA CAATTATAGT ACAATAACAA ACACAAAAAC AATACAGACG CTACTACTCA ATTCACACTG CCTTACATCC

- 513 TGAATTTAGA AGATTCACTG ATAAAGGAAT GAAAGAATTC TTATACCTAT TCAGGTATCT TTTCCTATAT TTAACCGGCA CCCTATAGCG TCTACCTGAA

- 413 GGGAGTAGTT CATATTCAGG GTATAGGACA TGGGAGGGGT CTGCTAGAAT TGTATTATAG ACTGTATGGC CAAAGTTTCA TTGTCAAAAT GTTTTAAACG

- 313 TGTGTACCCA TTGTTTATTT TGCTTATATA GTTATGTGGA CTAGGTTAGG CTATTTTGCA CCCACAAATC ACCATTCCCC CAAAAGAATA ACTAAATCAA
                                                           VDR
- 213 AGCATATAAA AGCAGTGTGG TTTACTTTAA TTGTGTGTTT CTGTTTTGTG CGTTTTTATT GTCTGGTGTT AAAGTCTCTA ATTCATCCAA ATGGCTCTTT

- 113 CTTTTCCAGA ATGTACCGTC GATCACTACA AAGATCAAGT CCACAATGGA TGTCTCTGTC TGTGTAGCCA CCCAAAGCCA ATGCAAATCT TTGACTGATA
        STAT3                                                                          CAAT-box
-  13 TTAGTATATG TAGctaacag ttgcttttta cctcctgcta cttttcattt gtggagatag acagggtgta gattgatttt ctaactcgtc ttctttattt
       TATA-box
+  88 ctttggatat ttatttattt attaagaaga aatacctcaa tagtctgacc tagtttgatt cacaacaggc caatagtgca tttactctcc agcaatggaa

+ 188 tccactacac tagcatccct tgcgactgtt accatg
```

(B)

```
-1804 GGTTTACTGT TTGACGCCAC CATCAACCGA AGACGACGGT CCCGCCCTCA CAGTCCAGTG ATATATGTCT TGCGTGGACA AGCAGGAACT CATCATCTGA
           ATF4
-1704 AGCCGGCTAA AAGCGGTTTA TTGCGAATTT TTATTTTATC AAGGACTCAA AATAATCAGC AGTGTCTTGT TTGTAGCTTT AAGCTTTGAA TTTACTTTAT
                         CEBPa
-1604 TTAACCGACG TTAGTGCTTA AGAAAGAGCG AGAGCATTTA TCGTGTCTTA CCCCGCGCGC GCGGAAAGCG TCTACGGGAA GTTGACTGGG CGGGCTACTG

-1504 TGTCAAAAAT ATCTAAATCG ATATTTATCG ACAGTTTTCT TTGAGTGGTG AGTAAAAATA TTTTTTCTTA TTCTATTGGT ACTGAGTGGT ATTTGTCGTA

-1404 CTTTCTCATC GACGTTTTTT TTCTTTATTT GTGAACGGCT AGAATGAGCG CGAGCTCGTG GCTTTGTCAG CTGACTCGAG GTGGTCGAAT GTGGAAAAAA
                                                             SREBP1
-1304 AATCATACGT ATCTAAGATT AGTAAATAAT ACGGTGTCAC CGATAAATGT TGTTTATATG TATAAAATAT TTATTTTGTG GTTATTTTGG AATGTTTTAA

-1204 TTAAGTACTT ATGATTTATA ACGTCATATT AGCTGTTAGC GGGTGTGAAT GAATGACGAC ACGTGTAGTG CGAAGCCGGC GCGCACAGAA AAGATAAATA
                       SREBP1                        ATF4
-1114 TCGCCTTGTT AACCTCAAAA GCCTAACAAA AATTGTTCTA TTTAAGTAGT GTACTATTTA ATTTTCATTA AAAAAATAGC ACCGAATCTA TAACGGCTTA

-1014 AAAACCGACG AATTAGGGTT GTTCGTGAGT GATTTGGTTC CTCAAGCTAA CGTTAGCGAG GAAGTGCGTG TCTAGCGCGA GCGGGGAGAA CTGGTTGGAA

-914  CAGCCCTACA GTGACAGACA GTCAAACGCG TTTCATCATT TATTCTCTGC GTTTTCTTTG CCAATAAAGG CTGCATTGCC GGAATGTACT GGAAAGCCAC
                                    CEBPa
-814  ATGACACGGA TGCGCTGGCA GCTTAATTTG AATGCAAGTT TCTATTTGTT GTCAAATAGC TTAAAATATT AATCAAAGTG GTGCGTTCTC GGGCTTGTAA

-714  AACAAACTAG TCCGATTGGC TATTTAGTAT TACGTTTTAT TACTTTGATT GTTTTATTAC TTGATGATTG TAGTCAAAAA GCCAAATAGA CATTGGTTTT

-614  AGTTCATGGA TAAGCGGATG GCATATATAT GTATTTTTAA AAGTTTTTGT TGTATGATCA GGCATGCATT CATGCTGTCT GTTCTCGATG CGCCCCCTTT
       VDR                                         NRF2
-514  CTATACAGTG AGAGTTTTCT AGAGACCTTC AGCTCCCCTC TCTGGCAAAT AAAGTACTGA TAATACTGCT TTAGTGTGTA AGAGTAGACT TATGTCTAAG

-414  TTCTGGCCCC TCTGCAGTGA AATTGTTGAG TAACACGGGA ACCTTCCCAC TTTCTCCTCT TTTTTTTTTT TTTTTTTTCA GGAATGAGCT ACCATTCACC
                              NRF2
-314  CATCGTGTCT GTTGCACAAT GGATGTCCGC TCTGTCTGCC TAATTGCCCA AGCCAAAACA AACTGTAACA GATCTAGTCT ATTCAGAGGA TAGCAATAGG
             CEBPa                             CEBPa
-214  CCCTCACTCC ACCCTCTCCC TTAACGTTAC ACATCAGTTT AGAGCAGAGC AGTGCAGATT CTCTCTCTAG TGTCTGAATT GCTTTGATTT GCTGGTTTAG
        SREBP1
-114  TGAAGGGCGA AGGTAGGTCT GAGGCAGAAA ACCGATTTAG AAACACTAGT ACCTCAATTT TCTGGGGATA AAATATCCAG TGTGCTGCTG CTAGAATTAT
                                         CEBPa                                                  TATA-box/TBP
-4    TTATacatat ttggttcttt tccctgtaag attgtgaagt taatttgttt ctgtataact tgaaaaccat ctgcgcacaa tggtatcgac aactttggcc

+97   acgataacgc ttttgagcac ccttgcaggg tatgctgcaa tcaataca
```

(C)

```
-1775 GGTCTGTGGT GAGAACGCTC TGTAATTTTA CAGGTGTTGG TTTTGACGTC ACTATATGAT CGTATACTGT GTCTTTGTAA TGCTTTAAAG TCCATTCAGT
         SREBP1
-1675 CATCAGCTGT TGACTGATGG TCTAAATACC GTACTGTCCC CTTGTGCTAT GGGATCAGTC GTTCTGATAA ACAGGACTGA TCAGATTTCA CCAAGCACAT

-1575 AGTGGCCCAT TTACAGACCT GCCGTTTTCC AATGAATTTG TCCCACTAAG ATTCTTAGAG TGGGGAAAGC AGGAAGCGAG GAGGCTGTAA GCAGGTATTA
                                 CEBPa
-1475 GGGGCTGCGA CTCTGCAGTG TTGCACAGTG CAGCTGGAGC ACGACTCGCT GTACTGCTTC GCCTCGGTGG GAGACTCAAA CCTGAAACGA AGCCAAGAAG
              NRF2
-1375 CCGAGAGGAG GTGTGAAGCA CGAGTGATTG TTTTGCAGTG CTTACTGCGC TGTTACATTC TGTTATGGAC GTATACTGTG TTAGTCACAT TGTGTCTTGG

-1275 TCACATCTGA ACGCTTCGCC CTTGGTGTCA TAGTGTAAGG TGGCCAGTTA ATGTAAGTGA TCAAGTGCTT TTCTTTCTGT TTTATGTAAT GCTATTTAAT

-1175 ATCTGATTTC AAACCTTGAA ATCCCATGTC TTTTGTTCAA TACATCTTTA TTGTACTTAC AAAAAAATAT AGCAAATTTT CATAATTTAA TTTTAGCATG
         VDR                CEBPa                        VDR                        ATF4
-1075 TCTGTATGGC ACCTCCTAAA CATGGTTATT TTGGTCTGTC TCACTTCAAA GTTCTTGACT GACTCTTTCC AACGATTAAA ATCATACAAT AACGTCATAA
                                                                                                  SREBP1
- 975 TATCATCATA ATCTTCACAT ACATATTCTT TTGAAATATT CAATTCATTG TAGTCAAAAC AAATTTCTGA TTATTAATCC TGTTACCACA TGATTTTCCC
                                   CEBPa
- 875 CCTTATTCCA AACAATGTAG CATTTCTCTA CATTTTTATT TGGACTTTCT GAGTGGCAAA TATTCAAACC TGTTACGCAT TCTGGCATGT TTATTTATTT

- 775 ATTTATTTAT TTGTAATTAT GAATTTTGTC TTTTAAAATG TTTATTACTA GAAAAAAACA CTAGATCAGT GTTTTAAATG TATTTATATG TCCTTACTCC

- 675 TAAGGTTGAA TTCTTGAGGT TTATAGTAAC CACTTTCTTG AGGATAACCA TATAACATTC CATTCCATTC CATATGTGAA AAGTTGACTT TTTCACATAT

- 575 TTTTCTTTTA TTTATGGAAA ATAACTACAA ACTGACATTT AGTTCCTATA GATAGCCCTT CCTGAGTGGT AATTTACCAC CCTGTTACTC ATTCATATTC
                 STAT3
- 475 ATTTTTATTT AATGTTTATT ACTAGAAAAA ACAAAACAAA ACTTGGATCA GTGTTTTAAA TGTGTCCCAA ACTTTTTTTT TTTTTTTTTT AATAATCAAC

- 375 ATTGTCTGAA GGATAAGCAG CACCTCTTTT TACTGACCAT TTGTTGATGT TTCTGCCTCA TTTTTGTACT TTATATGTGG AAAATTACCC TAATCCTGTA

- 275 TTTTAATTTT TTTCATGTCC TATCTCTCAC GTTCCTTGAT GTCCCCGAAA ACTTTTCCAT TTCCCATGTT TTTTTGGGCT ACATTACGCA GTCTATAAGG

- 175 AAAGTGTGAA TACTGTGTAA TGATGTCCTT TTCTCTCTTG TTTTGACAGC TGAAGGCGTC TTTGTGGACA GGACACTTAG AGGGCCAAAC AATGGAATGC
                                                                                           CAAT-box
- 75  TCTCTCCTGC CTACACTGCT CAGCACTCTA CAGACTGAGG TTAAACGGCT TAGTTAGCAG TGTACCTCTC CCCCAagaac tcttttagcc tggggaaagt
          Sp1                                                          Sp1
+ 26  tgggagaaat aaaaaccaag cgaaggagaa tttccttcat agtacagcac cttactccag ttctttgtag ttgtttggga ttcatttgta agctttgcgt

+126  gaacaaaagc cataatggaa acggaaacgt acctgtggat ggtcgtgctt ggcttcatta tagccttcat cctggcattt tcagtg
```

Figure S1. Nucleotide sequence of the 5′-flanking region of the *slc20a1a* (**A**), *slc20a1b* (**B**) and *slc20a2* (**C**) gene. Numbers are relative to the transcription start site (+1). The upstream sequences of transcription start site are in the capital letters, and the downstream sequences of transcription start site are in lowercase letters. The putative transcription factor binding sites are underlined.

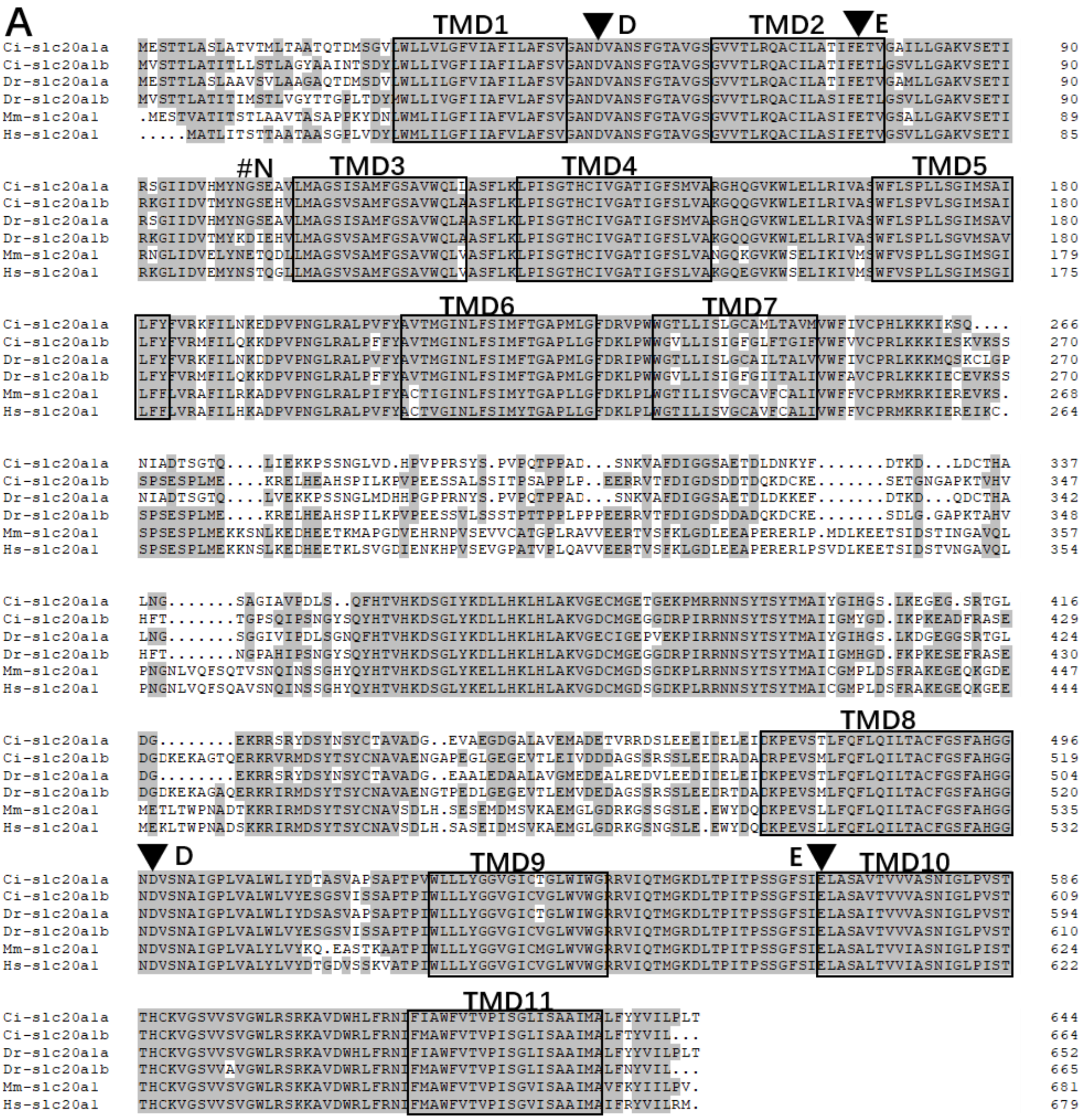
A
TMD1
D
TMD2
E
#N
TMD3
TMD4
TMD5
TMD6
TMD7
TMD8
D
TMD9
E
TMD10
TMD11
Ci-slc20a1a
Ci-slc20a1b
Dr-slc20a1a
Dr-slc20a1b
Mm-slc20a1
Hs-slc20a1

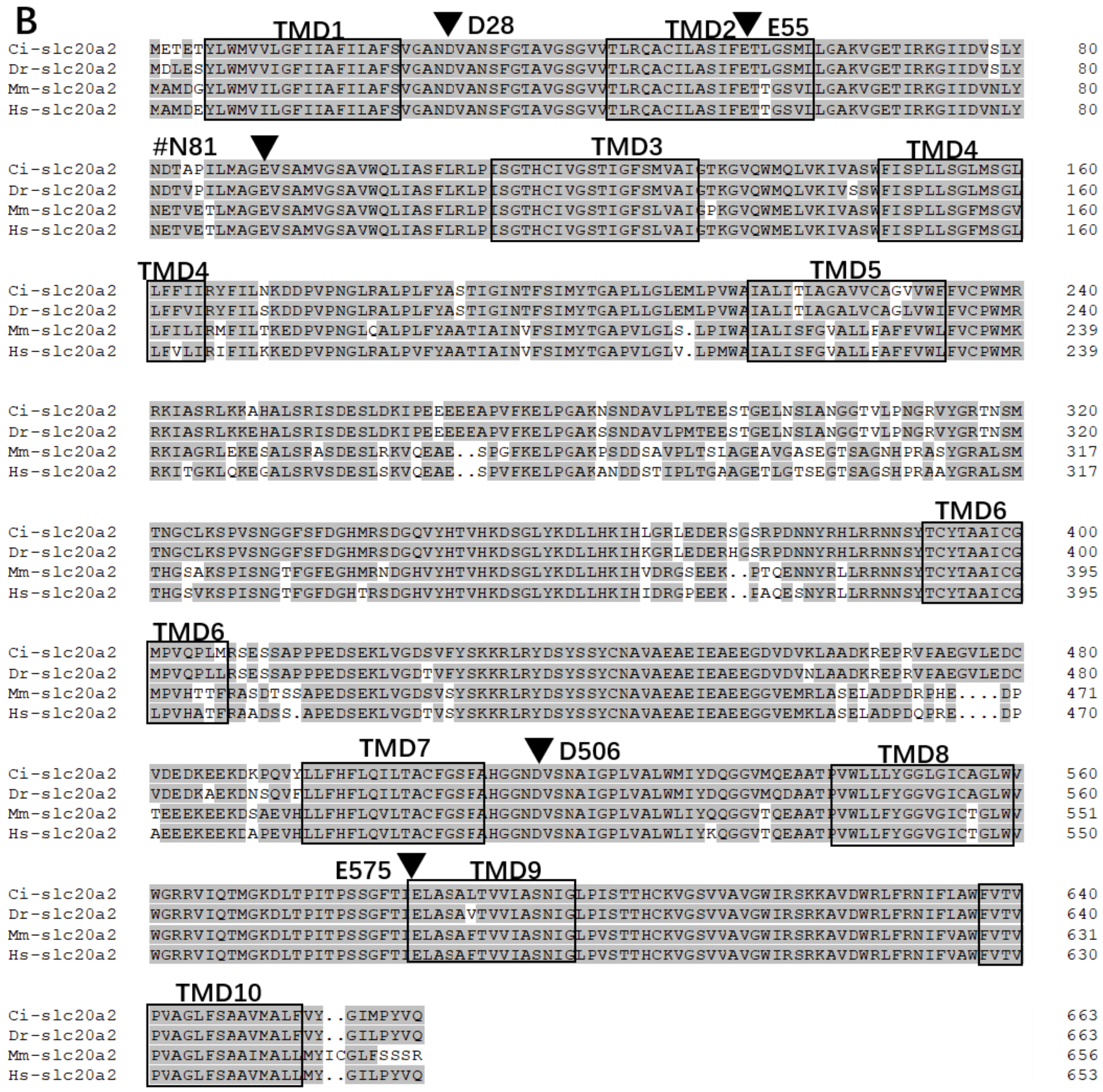

Figure S2. (**A**,**B**)Alignment of *Ctenopharyngodon Idella (Ci)* SLC20s amino acid sequences with *Danio rerio(Dr), Mus musculus (Mm)*, and *Homo sapiens (Hs)*. The identical residues were shaded with dark gray. The predicted transmembrane domains (TMDs) were outlined by boxes. Residues below triangle symbol represent the conserved acidic residues important for transport function. Residues below hash symbol (#) represent N-glycosylated site.
